# Lyme Disease with Erythema Migrans and Seventh Nerve Palsy in an African-American Man

**DOI:** 10.7759/cureus.6509

**Published:** 2019-12-30

**Authors:** Rebekah Dennison, Cheryl Novak, Alison Rebman, Arun Venkatesan, John Aucott

**Affiliations:** 1 Department of Medicine, Johns Hopkins University, Baltimore, USA; 2 Department of Medicine, Johns Hopkins University, Lutherville, USA; 3 Department of Neurology, Johns Hopkins University, Baltimore, USA

**Keywords:** lyme disease, seventh nerve palsy, african-american, erythema migrans, post-treatment lyme disease syndrome

## Abstract

An African-American man in his 30s presented following seven weeks of symptoms including an initial febrile illness with a rash followed by onset of fatigue, facial weakness, daily headaches, neck pain, leg numbness, hyperacusis, and photosensitivity. Over the seven weeks, he had several evaluations and was treated for cellulitis and facial swelling before ultimately being diagnosed and treated for Lyme disease with seventh nerve palsy and meningitis. His symptoms failed to completely resolve after treatment, and he was diagnosed with post-treatment Lyme disease syndrome (PTLDS) due to ongoing symptoms which lasted for more than six months after treatment.

Delayed diagnosis increases the risk of PTLDS and other long-term complications from Lyme disease. Provider awareness of Lyme disease risk factors, common neurologic and other presentations, and racial differences in diagnostic findings such as the skin rash can improve care by achieving earlier, accurate diagnoses and reduce risk of PTLDS.

## Introduction

Lyme disease is a multistage, tick-borne infectious disease caused by the spirochete Borrelia burgdorferi [1]. Early localized infection is characterized by an erythema migrans (EM) skin lesion around the tick bite, often accompanied by non-specific symptoms of fever, chills, fatigue, malaise, or arthralgia. In about 20% of cases, EM is not present or is not recognized [2]. EM lacks the typical bull's-eye appearance in approximately 80% of individuals in the endemic United States. Disseminated infection may involve the cardiac, rheumatologic, neurologic, or other organ systems, with widely varying symptoms depending on the organ system involved. Infection of the nervous system, sometimes referred to as Lyme neuroborreliosis (LNB), may result in meningitis, seventh nerve palsy, radiculopathy leading to peripheral numbness, tingling, or weakness [3]. Cranial nerve palsy accounts for up to half of LNB cases [4].

Prognosis is generally very good for patients who receive antibiotic treatment for Lyme disease, especially if treated in the early localized stage [5]. After the treatment, a subset of patients continue to experience symptoms or develop new symptoms of fatigue, cognitive difficulties, or musculoskeletal pain that can persist for more than six months. If no other underlying cause can be identified, these symptoms can be termed post-treatment Lyme disease syndrome (PTLDS) [6].

Differences by race in Lyme disease incidence in the United States have been reported, with White populations noted to have a disproportionately high incidence of the infection. This variation is usually attributed to area of residence; minority populations tend to be underrepresented in Lyme disease-endemic areas [7,8]. However, in one study in a rural endemic area with a substantial African-American population, Whites were more likely to have EM whereas African Americans were more likely to have late Lyme arthritis, suggesting that racial disparities may also be due to other contributing sociologic factors [8]. Overall, few studies have sought to investigate whether racial or other demographic factors influence patient care or outcomes in Lyme disease [7-9]. 

We report a case of delayed diagnosis of Lyme disease in an African-American patient. Misdiagnoses of Lyme disease-related EM and seventh nerve palsy led to delays in effective therapy and worsening of symptoms. The patient’s symptoms did not fully resolve and continued to negatively impact his quality of life several months after treatment. This case raises questions about diagnosis and care of patients from underrepresented populations, highlighting the need for additional studies investigating health disparities in Lyme disease diagnosis and management.

## Case presentation

**Figure 1:**
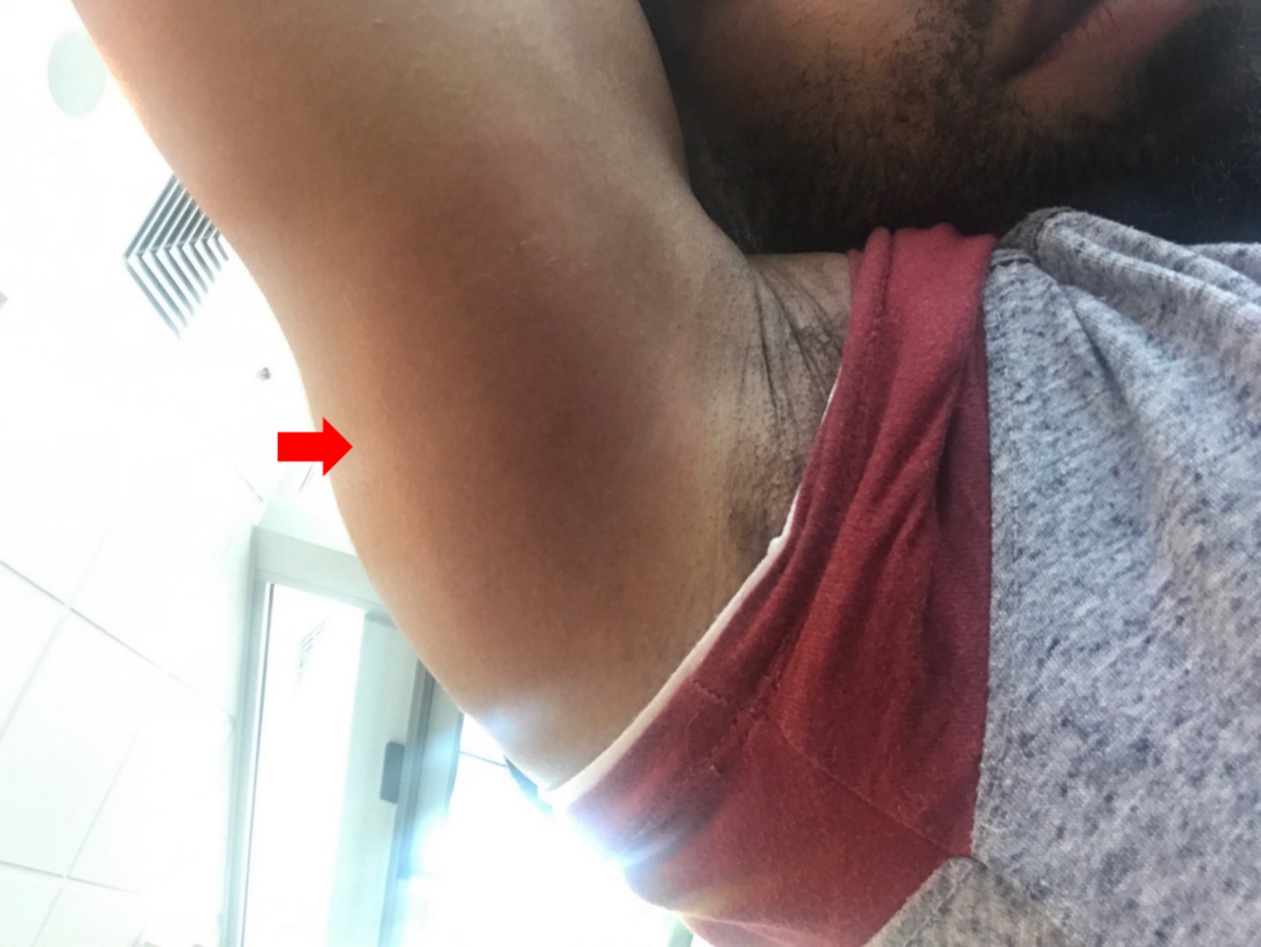
Erythema migrans (EM), right underarm

A 32-year-old African-American man initially presented for evaluation with a possible “bug bite” and an associated oval, red skin lesion on his posterior proximal arm, chills, and fatigue (Figure 1).

**Figure 2:**
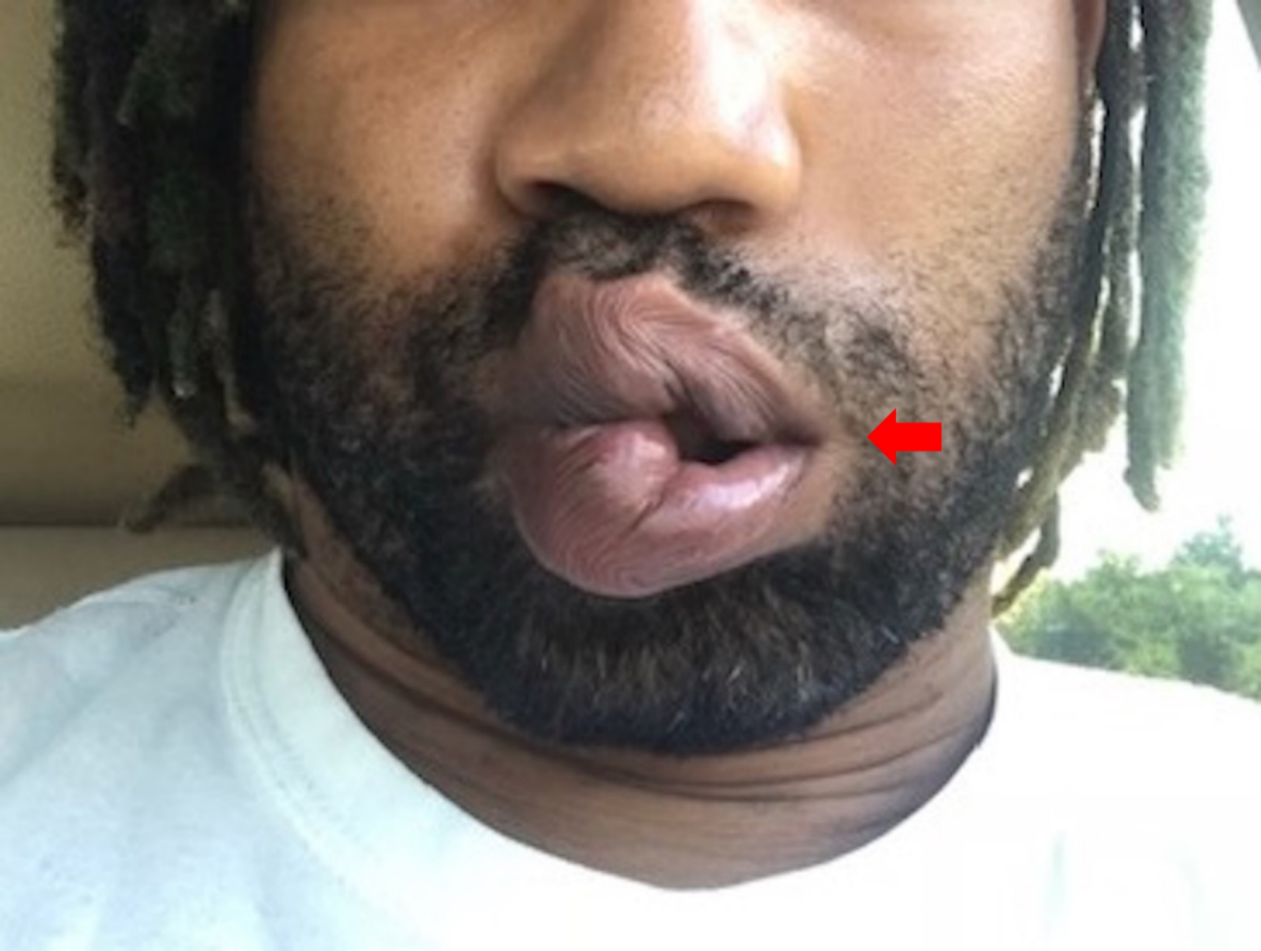
Left facial palsy

A resident of a suburban community in a major east coast metropolitan city had been camping with his children two weeks before the onset of his symptoms. He was diagnosed with cellulitis and treated with a 10-day course of cephalexin. Twenty-four days later, he developed facial weakness (Figure 2), neck pain, headache, irritability, mood swings, and left leg numbness. He was diagnosed with facial swelling based on the appearance of his face and lips and treated with prednisone 60 mg for a presumed allergic reaction. Three days later, he presented to the emergency room and was diagnosed with possible Lyme disease versus idiopathic Bell’s palsy and started on doxycycline and acyclovir. Two days later, he was notified of a positive Lyme serology with positive ELISA (enzyme-linked immunosorbent assay), IgM (immunoglobulin M), and IgG (immunoglobulin G) immunoblot (>5 bands).

Two days after completing a 10-day course of doxycycline, the rash had resolved but he continued to experience fatigue, arthralgia, myalgias, left leg numbness, daily headache and memory problems, and irritability with mood swings. A repeat emergency department evaluation diagnosed muscle tenderness without further treatment.

The patient presented to our clinic seven weeks after the onset of illness. At this point, he had no improvement in his symptoms and had developed new-onset hyperacusis (left ear) and photosensitivity. The physical exam was notable for a left seventh nerve palsy, left shoulder shrug weakness, and left upper and lower extremity weakness.

The patient was sent to the emergency room for evaluation of possible meningitis. Brain computed tomography scan without contrast was unremarkable. Lumbar puncture showed slightly elevated white blood cell count (8 cells/mm^3^) and unremarkable red blood cell count (9 cells/mm^3^) in tube 1; in tube 4, slightly elevated white blood cell count (7 cells/mm^3^), unremarkable red blood cell count (1 cell/mm^3^), normal protein levels (29.5 mg/dL), normal glucose (62 mg/dL), negative Gram stain and culture, and positive Borrelia burgdorferi immunoblot with IgG band (18 kD) and IgM band (39 kD). The presentation and spinal fluid results are suggestive of a mild aseptic meningitis. Viral infections such as enterovirus and West Nile virus are the most common causes of aseptic meningitis in the United States. Powassan virus, transmitted by the same tick that carries Borrelia burgdorferi, can also cause aseptic meningitis. Unlike typical bacterial infections that present with highly elevated cerebrospinal white blood cell counts with a neutrophil predominance, Lyme disease-associated meningitis typically presents with mild white blood cell elevation with a lymphocyte predominance [10]. Moreover, our patient’s infection may have partially resolved by the time of lumbar puncture due to previous antibiotic treatment. Our patient’s positive Lyme serology, combined with cerebrospinal fluid analysis that is consistent with Lyme disease-associated aseptic meningitis, supports the diagnosis of LNB. The patient was started on a 21-day course of intravenous ceftriaxone for LNB with seventh nerve palsy and meningitis.

Upon completion of intravenous ceftriaxone therapy, the patient had persistent headaches and little improvement in his facial palsy. T1 and T2 MRI sequences showed normal exam of the brain parenchyma and of the cerebrospinal spaces. Following gadolinium administration, enhancement was observed in the labyrinthine, tympanic, and descending/mastoid segments of the left facial nerve compared to these segments of the right facial nerve, as well as at least the proximal segments of the left seventh nerve within the parotid gland.

Four weeks after completion of intravenous ceftriaxone, the patient was seen by a neurologist for evaluation of continued fatigue, headache, facial weakness, and extremity numbness. On re-evaluation, the physical exam showed that he was alert and oriented to person, place, and time. There was decreased facial muscle activation in the lower portion of the left face and mild weakness with left eye closure and forehead wrinkling. Motor strength was 5/5 throughout, except the left lower extremity which was 4+/5 throughout, proximal and distal. There was no sensory level to pinprick or vibration; however, there was decreased pinprick and temperature circumferentially in the right arm. Electromyogram test and nerve conduction study were ordered but were not performed.

Eight months after completion of therapy for Lyme disease, the patient has ongoing symptoms of fatigue, muscle and lower back pain, and cognitive difficulties. His seventh nerve palsy persists with partial improvement while the hyperacusis has improved. His health-related functioning and quality of life continue to be limited. He has been diagnosed with PTLDS after an extension evaluation has failed to reveal any other cause of his ongoing symptoms.

## Discussion

Delayed or missed diagnosis of Lyme disease, including LNB, is an ongoing challenge due in part to the wide range of possible presenting symptoms [11]. Our patient experienced misdiagnoses that resulted in delayed treatment and progression of Lyme disease. His EM was initially misattributed to cellulitis, leading to a treatment delay of several weeks. EM can appear as a hallmark “bull’s-eye” or target-shaped lesion, a uniform red or bluish-red lesion, multiple lesions, or can more rarely have a small, central blister [5,12]. EM can be difficult to recognize when the stereotypical target lesion is absent, or when the lesion is in an area that is difficult to see, as was the case for our patient. We hypothesize that the rash may also be less visible on the skin of people of color and that providers in non-endemic areas may have less experience or education diagnosing EM in patients of color [7]. Very few examples of EM rashes have been published in the literature, and while attempts have been made to expand educational materials related to dermatological findings in patients with deeply pigmented skin, ongoing concerns remain regarding provider training for diagnosis and management of skin diseases in people of color [13,14]. Underexposure to examples of disease presentations in patients of color, coupled with a lower incidence of Lyme disease among African-Americans, may have contributed to misdiagnosis of both our patient’s rash and his facial swelling.

When our patient developed unilateral facial weakness 24 days after his initial illness (Figure 2), he was treated for facial swelling, resulting in a further treatment delay. Neurologic involvement occurs in 12%-14% of Lyme disease cases and as many as half of these are cases of cranial nerve palsy [4,5]. Moreover, Lyme disease is one of the most common causes of seventh nerve palsy in Lyme-endemic regions, where it accounts for up to 25% of facial palsy cases in adults and 34% or more of cases in children [15]. Paralysis may be unilateral or bilateral. The co-occurrence of EM or systemic symptoms such as fever, headache, or myalgias can help diagnose seventh nerve palsy due to Lyme disease. Neurologic symptoms generally occur within a few weeks to months after the initial febrile illness, as occurred in our case.

Our patient received antibiotic treatment on day 27 of his illness. At this time, his Lyme disease serology was positive. Serology can be of limited use in very early Lyme diagnosis, as antibodies may not appear in samples for several weeks following infection [4]. For our patient, the combination of positive ELISA, IgM, and IgG confirmed the clinical diagnosis of acute infection.

Seven weeks after the start of our patient’s illness, lumbar puncture was performed for evaluation of possible meningitis. In cases of cranial nerve palsy, the role of lumbar puncture to evaluate for coexisting meningitis is debated [3,4]. Parenteral antibiotic treatment may be considered for patients presenting initially with central nervous system involvement, although studies have shown comparable results with oral antibiotics [3,4]. The prognosis for seventh nerve palsy is generally excellent with oral antibiotics, but in rare cases such as ours there may be incomplete recovery. It is unknown if delay in therapy is a risk for poor recovery of seventh nerve function, corticosteroid exposure during acute infection has been shown to be associated with worse long-term facial function outcomes [16]. Our patient was retreated with intravenous ceftriaxone.

In about 10%-20% of Lyme disease cases, new or persistent symptoms may continue for more than six months after initial treatment, as was the case for our patient. Such cases are classified according to the Infectious Diseases Society of America’s case definition as PTLDS when no other underlying cause of symptoms can be identified. PTLDS includes a constellation of symptoms such as persistent fatigue, cognitive dysfunction, and ongoing joint and muscle pains that may be intermittent or constant [6]. While the mechanism of PTLDS is unknown, known risk factors include a delay in initial diagnosis, numerous and/or severe symptoms, and presence of neurologic involvement at the time of initial treatment [6]. Our patient experienced diagnostic delay and had several severe symptoms, including neurologic symptoms, by the time of initial antibiotic treatment. Ideal long-term management of PTLDS remains uncertain, and for some patients, PTLDS continues to impact quality of life for years [17].

Our patient’s initial illness displayed several common features of Lyme disease, yet he did not receive testing or treatment for Lyme disease until his third medical visit, 27 days after the start of symptoms, nor was he evaluated for LNB despite the increasing severity of his neurologic symptoms. It is unknown whether this specific case is a direct result of implicit bias; however, it does highlight both the ongoing relevance of early misdiagnosis of Lyme disease and, more broadly, how medical care is shaped by demographic factors such as race, class, and gender [18]. It is well established that implicit bias is widespread in healthcare, but few studies have investigated Lyme disease-related health disparities in the United States, where reports of Lyme disease in patients of color are disproportionately less common than in White patients [19].

Fix et al. have described differences in case reporting between White and African-American patients in a rural endemic area of Maryland with a substantial African-American population [8]. Notably, incidence of EM was lower among African-American patients, whereas incidence of late-Lyme arthritis associated with ongoing infection was higher than in White patients [8]. Together, this suggests that in Lyme disease-endemic areas, African-American patients may be at higher risk for missed or late diagnosis, challenging the common explanation for variation in incidence rates based solely on area of residence [7,8]. This finding, consistent with our patient’s late diagnosis, should prompt investigation of why such delays or misdiagnoses occur.

Demographic factors can influence many aspects of health care access, delivery, and management, including trust between patients and providers; patient education about disease prevention, symptoms, and when to seek care; insurance status; income, including cost and impact; and the potential roles of poverty, discrimination, and stigma [7-9,14,20]. Given shifting populations in the United States, and the increased risk of long-term complications from Lyme disease after delayed diagnosis, there is great need for further study to understand how these types of factors may present obstacles to early diagnosis and treatment of Lyme disease [6,14].

This case points out difficulties in diagnosing Lyme disease-associated EM and LNB, which we posit may be of increasing importance for African-Americans and other patients of color. Delayed diagnosis can have serious complications, and there is a need for further investigation regarding whether patients of color are at increased risk for delayed diagnosis as well as broader research on how demographic factors may influence Lyme disease diagnosis and management.

## Conclusions

We describe the case of an African-American male who presented with common early, and later disseminated neurologic, manifestations of Lyme disease who was misdiagnosed over a period of seven weeks. Patients with a delayed initial diagnosis, more numerous or more severe initial symptoms, or neurologic involvement are at a higher risk for developing PTLDS. More studies are needed to investigate the role of racial and other demographic factors in Lyme disease diagnosis, treatment, and outcomes. Careful physical evaluation combined with recognition of Lyme disease risk factors, presentation types, and possible disparities in care can help achieve early, accurate diagnoses and reduce risk for ongoing complications related to Lyme disease.
